# Second Chances in Learning: Does a Resit Prospect Lower Study-Time Investments on a First Test?

**DOI:** 10.5334/joc.196

**Published:** 2022-01-06

**Authors:** Rob Nijenkamp, Mark R. Nieuwenstein, Ritske de Jong, Monicque M. Lorist

**Affiliations:** 1 Department of Experimental Psychology, University of Groningen, Groningen, NL; 2 Research School of Behavioural and Cognitive Neurosciences, University of Groningen, Groningen, NL; 3 Cognitive Neuroscience Center, Department of Biomedical Sciences of Cells and Systems, University Medical Center Groningen, University of Groningen, NL

**Keywords:** resit exams, study-time allocation, advance study-time planning, learning, exam taking

## Abstract

Previous studies have shown that the prospect of a resit opportunity lowers hypothetical study-time investments for a first exam, as compared to a single-chance exam (i.e., the resit effect). The present paper describes a first experiment in which we aimed to generalize this effect from hypothetical study-time investments to a learning task allowing for the optimization of actual study-time investments while participants studied pairs of pseudowords for a subsequent multiple-choice test, given either a single chance or two chances to pass. Against our expectations, the results of the experiment showed no resit effect for the amount of actual time participants spent studying the materials in the experimental learning task. To better allow for the optimization of study-time investments, the learning task was adapted for a second experiment to include an indication of passing probability. These results, however, also did not show a resit effect. A third experiment addressed whether it was the investment of actual time that led to this absence of a resit effect with the learning task. The results suggested, however, that it was most likely the lack of a priori deliberation that caused this absence of the effect. Taken together with findings from a fourth questionnaire study showing that students seem to take a resit prospect into account by indicating they would have studied more for an exam if the option to resit would not have been available, these findings lead us to argue that a resit prospect may primarily affect advance study-time allocation decisions.

## Introduction

Resit exams are a type of exam used in certain educational systems that adhere to the learning for mastery system (Bloom, 1968; Carroll, 1963; Kulik et al., 1990). They offer students the opportunity to take an examination a second time after failing the first exam attempt in order to have a second chance at showing mastery of course contents, and thus offer a second chance at passing a course without having to re-do the course as a whole. Concerns have been expressed in the literature, that these resit exams may result in grade inflation, due to potential undesirable effects of the prospect of such resits on student learning and, more specifically, on students’ strategies regarding study-time investment and test taking (Al-Bayatti & Jones, 2003; Centre for Education Research and Policy, 2012a, 2012b; see also Burr et al., 2018; McManus, 1992; Pell et al., 2009; Ricketts, 2010; Scott, 2012; Yocarini et al., 2018). However, empirical evidence for such potential adverse behavioral effects of resit exams is scarce. This may be one of the reasons for a pronounced lack of consensus across educational systems and institutions regarding the question of whether resit exams should be offered at all and, if so, what appropriate resit policies are.^1^

In recent work it has been found that the prospect of having a second chance to pass a simulated exam (i.e. a resit exam) indeed leads to a reduction of *hypothetical* study-time investment, as compared to only having a single chance to pass the simulated exam (Nijenkamp et al., 2016, 2018). Since these studies found this effect with the investment of only *hypothetical* study time, and the fundamental psychological mechanisms underlying the effects of a resit prospect are not yet precisely known, it is paramount to establish whether a similar resit effect would occur in a task requiring the investment of actual time on learning materials for a subsequent test. Therefore, the current paper reports the results of a controlled experimental study using a paired-associates learning task asking for the investment of actual time to study for and pass a few short multiple-choice tests. In a follow-up experiment this learning task was adapted to include an indication of passing probability to better allow for the optimization of study-time investments. Additionally, a third controlled experiment is discussed that assessed whether the resit effect still occurs with the investment of actual time using an adapted version of the study-time investment task used to initially observe the resit effect (Nijenkamp et al., 2016). Finally, the results of a questionnaire study, asking students whether they would have changed their study behavior if the option to resit a failed first exam would not have been available for two exams they made previously, are reported.

## Evidence for the Resit Effect

Despite the limited empirical evidence available on the effects of resit exams, there are some indications that they might indeed influence the way students prepare for exams if actual study-time investment is involved. In an experimental field study, for example, Grabe (1994) manipulated the resit policy for a course, by either allowing students a single opportunity to pass an exam, or three opportunities (i.e., two resit opportunities) with either the best or last grade of these opportunities counting towards the final grade. Grabe observed that student performance on the first out of three exam opportunities (with the best grade out of all attempts determining the final grade) was significantly lower than when a single exam opportunity was available. This finding suggests that, under some conditions, the opportunity to resit results in poorer preparation for the first exam opportunity.

Grabe (1994) argued that the resit effect could reflect a negative effect of the availability of second chances on students’ motivation to do well on the first examination attempt. In line with this reasoning, research has shown that students’ academic performance does indeed seem to be sensitive to differences in the specific rules making up assessment policies, possibly through motivational and self-regulatory factors (Elikai & Schuhmann, 2010; Johnson & Beck, 1988; Kickert et al., 2018, 2019). Further research has also shown that academic performance (Kickert et al., 2020) and study delays (Schmidt et al., 2021) might be affected by changes in resit policies specifically. Moreover, in another study it was reported that students, at least those who are impatient, tend to not exert sufficient effort to pass through the first exam and instead shift their study efforts to the resit exam (Non & Tempelaar, 2016). Assuming that the net invested study time for the resit exam would be similar to that invested for a single chance exam (as suggested by Nijenkamp et al., 2016), this would imply the presence of a resit effect in at least a subset of students.

In another line of research, poorer test performance in the first attempt has been explained using utility-maximization models (Kooreman, 2013b, 2013a; Michaelis & Schwanebeck, 2016; Nijenkamp et al., 2016; for a similar conceptualization, see also Wilbrink, 1980). Such models assume that rational students will seek to optimize a trade-off between the cost of investing study time, thereby gaining knowledge, and the probability of passing an exam. Accordingly, they generally predict that the optimal study-time investment for the first exam is lower when the option to resit is available. Nijenkamp and colleagues (2016, 2018), for example, used an extension of Kooreman’s (2013a, 2013b) mathematical model specifically modeling multiple-choice exams, by incorporating a well-established exponential learning function to relate the amount of acquired knowledge of course materials to invested study time. This model allowed for precise predictions for the effects of different resit policies on the optimal study-time investment, and these predictions were subjected to an empirical test using an investment task that required students to invest hypothetical study-time to pass a simulated multiple-choice exam. The results showed that, in close accordance with the model’s predictions, investments of hypothetical study time were lower for a simulated exam with resit opportunity than for a single exam opportunity (i.e., the resit effect). Furthermore, the magnitude of this resit effect was found to be positively correlated with the Cognitive Reflection Test (CRT; Frederick, 2005), which in turn has been shown to be correlated to performance on several indices of rational decision making (Toplak et al., 2011). Thus, the above results support the notion that resit opportunities could lower students’ preparation for an exam, and suggest that especially rational students may exhibit a resit effect.

An important consideration in interpreting the results of these earlier studies (Nijenkamp et al., 2016, 2018) is that students, and especially rational students, are capable of using the available information about the relationship between study time and passing probability to maximize expected utility in the condition without resit opportunity. Moreover, these studies demonstrate students’ cognitive abilities to appreciate and utilize the fact that the function relating overall expected utility to invested study time for the first exam is changed, often in quite subtle ways, by the availability of a resit opportunity under various resit policies and assumptions about the degrees of forgetting in between exams (for graphical illustrations of such changes, see Nijenkamp et al., 2016, 2018). This suggests that, at the very least, students must have appreciated the fact that availability of resit opportunities lowers the optimal amount of to-be-invested study time in the first attempt. Since they adapted their time investments in the first exam accordingly, and thereby accepted the lower passing probabilities related with lower study-time investments, this can also be taken to suggest that providing a resit opportunity could promote the use of risky study-time investment strategies.

While the previous findings demonstrate that students are capable of maximizing utility when investing hypothetical study time to pass a simulated exam under different resit policies, a potentially relevant limitation of the investment task used in the previous studies by Nijenkamp and colleagues (2016, 2018) is that participants were provided with precise and reliable information about passing probability as a function of invested study time. In real-life learning tasks, however, estimates of passing probability will likely not be given and students instead will have to rely on their own estimates. These estimates might be rather imprecise and biased, as they are based on judgments of learning during actual studying (Bjork et al., 2013; Metcalfe & Kornell, 2005), or on general experiences and beliefs regarding how passing probability varies as a function of study time for that type of task and expected type of exam. This does not mean, however, that students could not attempt to maximize expected utility using those imprecise estimates and as a result produce a resit effect.

## Experiment 1

As mentioned above, the investment task used to initially observe the resit effect did not require the investment of actual time on studying materials for a subsequent test. Therefore, the results of the studies by Nijenkamp and colleagues cannot be taken to imply the presence of resit effects in settings that do involve learning through the investment of actual time. Therefore, as a first step to test whether the resit effect, stemming from the optimization of a trade-off between the cost of investing time and the benefit of passing the exam or test, generalizes to a context requiring the investment of actual time on studying materials for a subsequent test, the present study extends the above line of investigation with the use of an experimentally tractable Paired-Associates Learning (PAL) task. The PAL task retained many of the mechanics of the investment task used in previous research (e.g., Nijenkamp et al., 2016). Specifically, the two tasks are similar in the sense that in both tasks participants were informed beforehand about the cost of investing study time and that they were incentivized to optimize the trade-off between the cost of investing study time and the probabilistic benefit of passing the exam in order to maximize a monetary pay-off. The crucial difference between the study-time investment task and the PAL task, however, is that participants in the PAL task had to invest, actual time studying material, rather than hypothetical study time, without having to indicate beforehand how much time they would want to invest.

Since study-time allocation has been found to be affected by aspects such as item difficulty (Son & Kornell, 2009; Son & Metcalfe, 2000; Thiede & Dunlosky, 1999), pseudowords were utilized in the PAL task rather than English words.^2^ Pseudowords are more uniform in nature and are without semantic content (see Mak & Twitchell, 2020). This absence of semantic information should increase item difficulty, and therefore facilitate strategic optimization behavior, as previous research has found that an increase in item difficulty triggers top-down strategic allocation of study time (Undorf & Ackerman, 2017). Moreover, the use of pseudowords should have reduced any influence or noise as a result of the specific randomized stimuli participants were exposed to, and allowed us to isolate any effects of a resit prospect on actual study-time allocation. Additionally, a surprise memory test was included at the end of the experiment to test whether any differences in study-time allocation as a result of having one or two chances to pass the test would also lead to a difference in the retention of the studied pseudoword pairs.

In previous studies using the study-time investment task (Nijenkamp et al., 2016, 2018) the goal was to simply pass the test to receive a small monetary bonus and, to allow them to maximize this bonus, participants were provided with the information necessary to approximate the shifting optimal study-time investments due to a resit prospect. Since the PAL task used in the current experiment retained many of these features, we hypothesize that participants will invest less time studying the pseudoword pairs for a test if a resit is available. We also expect the resit effect to be larger for participants scoring higher on the CRT (Frederick, 2005; Nijenkamp et al., 2016; Toplak et al., 2014). Furthermore, mirroring concerns expressed in the literature that providing resit exams could inflate grades, and therefore pass rates (Centre for Education Research and Policy, 2012a, 2012b), and in accordance with previous findings (Nijenkamp et al., 2016), we hypothesize that overall pass rates will be higher in the condition with a resit, while the average time invested per passed exam (regardless of passing through the first chance or resit) will be lower. Due to the exploratory nature of the surprise memory test, no specific hypotheses are made with regards to the possible outcome.

### Method

#### Participants

We conducted an a priori power analysis (Faul et al., 2007), assuming both a Type I and Type II error probability of .05 (i.e., Power = 0.95). Using the resit effect data from Experiment 1 by Nijenkamp and colleagues (2016; *M_NR_* = 6.22, *SD_NR_* = 0.53, *M_R1_* = 5.22, *SD_R1_* = 0.71, *r* = 0.58, *d_z_* = 1.69), the analysis based on the difference in means between two dependent matched pairs revealed a necessary sample size of 6 participants to attain sufficient statistical power to observe a resit effect. Since actual, rather than hypothetical, study-time investments might lead to more noisy data, we included a higher number of participants to ensure sufficient statistical power. Ultimately, 46 first-year Psychology students (31 female) from the University of Groningen participated in exchange for course credits during a single data collection period. Their ages ranged from 18 to 29 (*M =* 20.9, *SD* = 2.5). The study was approved by the Ethical Committee Psychology (15160-NE), and participants gave their written informed consent prior to starting the experiment.

#### Materials

The PAL task was programmed in MATLAB (MathWorks, 2017), using the Psychophysics Toolbox extensions (Brainard, 1997; Kleiner et al., 2007; Pelli, 1997), and run in a setting consisting of 10 computer set-ups enclosed by paperboard walls. The PAL task used pseudowords that were generated using the Wuggy software (Keuleers & Brysbaert, 2010), using the sub-syllabic English language module. The words used to generate the pseudowords consisted of 294 English language words randomly chosen from a list of words downloaded from the English Lexicon Project website (Balota et al., 2007), with a length of 5 characters, a log Hyperspace Analogue to Language (Lund & Burgess, 1996) frequency of 6 or more, and which did not end with an –s, due to the fact that these words consisted mostly of plural nouns.

#### Design and procedure

The task consisted of six tests (i.e., blocks) with or without a resit opportunity, for which participants had to spend time studying the accompanying materials. As the presence of a resit opportunity was a within-subjects manipulation, each participant studied for and made 3 tests with and 3 tests without the opportunity to resit a failed first-chance test. The presence of a resit opportunity alternated between tests, with the presence of a resit for the first test being counterbalanced across participants. Prior to studying the materials for each test, participants were told how many chances they had to pass that test and that they would have a maximum of five minutes to study twenty pseudoword pairs. The twenty pseudoword pairs that needed to be studied were chosen at random for each test for each participant from the 294 total available pseudowords, to avoid any bias in the study materials. Participants were instructed that they could cycle through the items and proceed to the test at any point in time, depending on their own judgment of whether they were ready to take the test. They were also informed that for some of the tests they would have a second chance to study and pass in case they failed to do so through the first chance, and that their aim in the experiment should be to pass each test by obtaining a minimum grade of 6 out of a maximum of 10. Before the experiment, participants were informed of the nature of the trade-off between spending time on studying and passing the test: if they spend less time on studying the materials while still passing the test, they would earn a higher monetary pay-off than when they would spend more time on studying.

**Figure 1:**
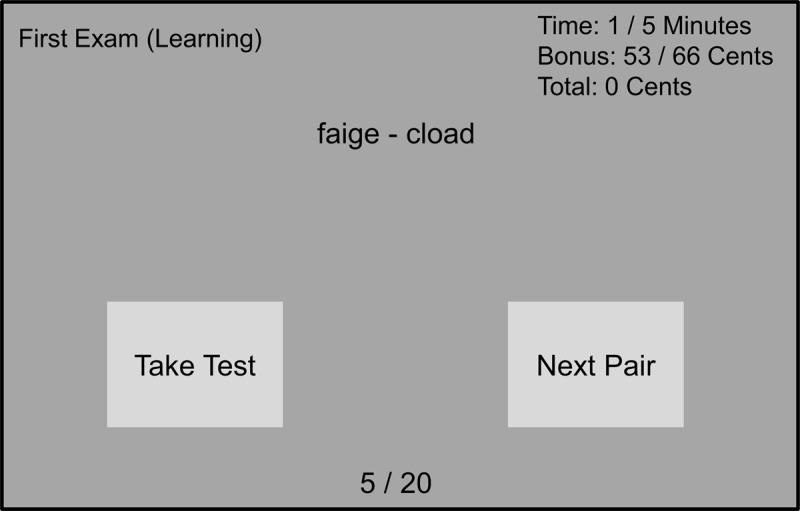
**Task display.** The task display as presented to participants during the study phase in Experiment 1. The display showed the pseudoword pair in the middle, an indication of the type of exam in the top left, an index indicating which word pair the participants was studying at the bottom, and the time, bonus, and total earnings counters at the top right. Additionally, the display included two light-gray buttons on which the participants could click at any point to either continue to the next pair, or take the test.

During the study phase, the pairs of pseudowords that needed to be learnt were shown one after the other in an order that was fixed for that test and that participant. Participants controlled the presentation of pairs using the mouse and they could re-study the pairs if they desired to do so. On the screen, it was shown how much time was left of the total of five minutes of available study time, as well as how many pairs the participant had viewed. Additionally, a decreasing counter showed how many cents participants could earn at any given moment during the learning phase if they moved on to and passed the test (***Figure 1***). In the test phase, participants were asked to complete ten multiple-choice questions based on ten pseudoword pairs that were randomly selected from all studied word pairs. Each question showed the first studied pseudo word of the pair, together with three answer alternatives for its associate. These alternatives included the correct answer, a foil chosen from the other pseudoword pairs presented as study materials for the same test, and a randomly chosen pseudo word from a list of unused words. After the test, the grade, which was equal to the number of correct answers, was presented. The criterion for passing the test was 6 correct answers. If a participant did not pass the test (i.e., grade ≤ 5), then they would lose a number of cents that was proportional to the amount of time that was invested during the learning phase. However, if a participant passed the test, then the outcome would be a gain of a number of cents that was proportional to the time not used for studying (i.e., the monetary bonus that was shown on the screen at the time the participant was studying and chose to take the test). The importance of this trade-off was explained to participants in the instructions to encourage them to optimize this trade-off between the costs of investing time on learning the pairs and the benefit of passing the test (i.e., gaining a monetary bonus). The maximum monetary bonus per test was set at 66 eurocents, and the amount they received at the end of the experiment was dependent on their performance (max €4).

For the tests in the condition with a resit opportunity, participants had two chances to pass each test. If they failed to pass the first test, participants went through the learning phase and test phase a second time. The procedure for the learning phase and the test phase of the first attempt (R1) of the tests with resit opportunity was the same as the procedure described above for the single-chance test (No Resit, or NR) scenario. For the resit opportunity (R2), participants again had five minutes to restudy the same material as for the first test. The test for R2, however, consisted of eight out of the ten pseudoword pairs that were not tested during R1, plus two randomly selected pairs that had also been tested in R1, and alternatively asked participants to choose the correct option for the first pseudo word of the studied pair after the second word was prompted. The remaining two untested pairs of the 20 studied pairs were used near the end of the experiment for the surprise memory test. For this test, participants were presented with pairs of pseudowords they studied in that combination previously, or pairs where the second pseudo word was randomly chosen from a list of previously unstudied words. There was a 50% chance that the presented pseudoword pair was as studied previously, or whether it was a new pairing of pseudowords. Participants were asked to indicate whether the pair had been presented in that combination during the study phase (‘correct’), or whether it was a new combination (‘incorrect’).

At the end of the experiment participants completed the Cognitive Reflection Test (CRT, 7-item version; Toplak et al., 2014). The CRT assesses the tendency to reflect on an intuitive, yet incorrect response alternative until the correct response is found. CRT scores have been found to be correlated with measures of rationality (Toplak et al., 2011).

#### Data analysis

We computed Bayes factors to assess the extent to which the data provided evidence in favor of or against our predictions (see Rouder et al., 2009). Bayes factors were calculated using the JASP software package (Love et al., 2019). In reporting the results of the Bayes factor analyses we adhered to Wetzels et al. (2011) for classifying the Bayes factors.

### Results

Comparing the average study-time investment in seconds for the first-chance (R1; *M* = 175.2, *SD* = 69.4) and single-chance exam (NR; *M* = 181.2, *SD* = 68.6; see ***Table 1***) a Bayesian one-sided paired samples t-test revealed there was anecdotal evidence (*BF*_10_ = 0.58) against the hypothesis that the time invested in R1 would be less than that invested in NR. In a subsequent analysis, we investigated whether the resit effect (i.e., study time NR minus study time R1) correlated with a participant’s CRT score. A one-sided Bayesian correlation analysis revealed there was anecdotal evidence against our hypothesis of a positive correlation (*r* = .13, *BF*_10_ = 0.41). In other words, there was no clear evidence distinguishing whether the resit effect was larger in magnitude for participants scoring higher on an index of rationality or not.

**Table 1:** Descriptive Statistics Experiment 1.


TEST	MEAN OVERALL STUDY TIME IN SECONDS (*SD*)	MEAN STUDY TIME PER WORD PAIR IN SECONDS (*SD*)	MEAN NUMBER OF WORD PAIRS VIEWED (*SD*)	MEAN GRADE (*SD*)	MEAN PROPORTION OF PASSED TESTS (*SD*)

**NR**	181.2 (68.6)	5.2 (3.3)	37.6 (13.0)	7.7 (1.7)	0.85 (0.27)

**R1**	175.2 (69.4)	5.4 (3.3)	35.8 (12.1)	7.5 (1.8)	0.82 (0.28)

**R2**	95.5 (71.4)	3.7 (2.9)	27.3 (13.0)	6.9 (1.9)	0.68 (0.43)


*Note*: Mean values, averaged over both passed and failed tests, for overall study time in seconds, study time per word pair in seconds, number of word pairs viewed (values higher than the total amount of included word pairs per test reflect the repeated studying of certain pairs), grade, and percentage of passed tests for NR (single chance), R1 (first test opportunity), and R2 (resit opportunity) in Experiment 1. Standard deviations are presented in parentheses.

For the analysis comparing the time invested per passed test, a one-sided paired samples t-test revealed strong evidence (*BF*_10_ = 0.10) against our hypothesis that the time invested per passed test would be lower in the overall resit condition (*M* = 193.2, *SD* = 68.9) than in the no-resit condition (*M* = 188.8, *SD* = 64.7), with participants investing marginally more time per passed test in the resit condition.^3^ Comparing the pass rates (i.e., the proportion of tests with a grade ≥ 6) between the resit and no-resit conditions through a one-sided paired samples t-test, we found substantial evidence (*BF*_10_ = 4.92) that pass rates were higher in the condition with resit (*M* = 0.94, *SD* = 0.17), than in the no-resit condition (*M* = 0.85, *SD* = 0.27).

As an exploratory analysis we also examined the results for the surprise memory test to determine whether the retention of the item pairs differed between the resit and no-resit conditions. A two-sided Bayesian paired samples t-test revealed there was substantial evidence in favor of a null effect (*BF*_10_ = 0.25), indicating no difference in the proportion of correctly identified word pairs that were learned during the experiment for the no-resit tests (*M* = 0.6, *SD* = 0.2) and the tests with resit opportunity (*M* = 0.7, *SD* = 0.2).

We also ran analyses on the performance on tests three through six, based on the idea that a resit effect might well depend on participants having gained some experience regarding the relationship between investing study time and the probability of passing the test. The results of this analysis, however, were similar to those based on all tests, presented above.

### Discussion

Except for the rather obvious finding that overall pass rates were higher in the resit condition than in the single test condition, our hypotheses were not confirmed. Most important, the resit effect observed in previous work using an investment task with hypothetical study-time investments (Nijenkamp et al., 2016) did not generalize to the PAL task utilizing actual time to study materials for a subsequent test used in the current experiment. Note that this is despite the fact that the average grade in the no-resit condition was 7.7 (out of 10), which is substantially higher than the minimum passing grade of 6, leaving seemingly ample room for a reduction in study time and the concomitant passing probability for R1 (i.e., a resit effect). A possible explanation for this discrepancy lies in the fact that in the previously-used investment task the precise relationship between study time and passing probability was visually displayed to participants, possibly aiding them in the optimization of their study-time investments, whereas no such explicit information was available to participants in the current experiment. We therefore adapted the PAL task in a follow-up experiment to include an indication of passing probability as an increasing function of the time spent learning the materials. With this addition, the information presented to participants in Experiment 2 more closely resembled that displayed in the study-time investment task, and might, as compared to Experiment 1, provide participants with additional information and motivation to optimize their study-time investment decisions and produce a resit effect.

## Experiment 2

**Figure 2:**
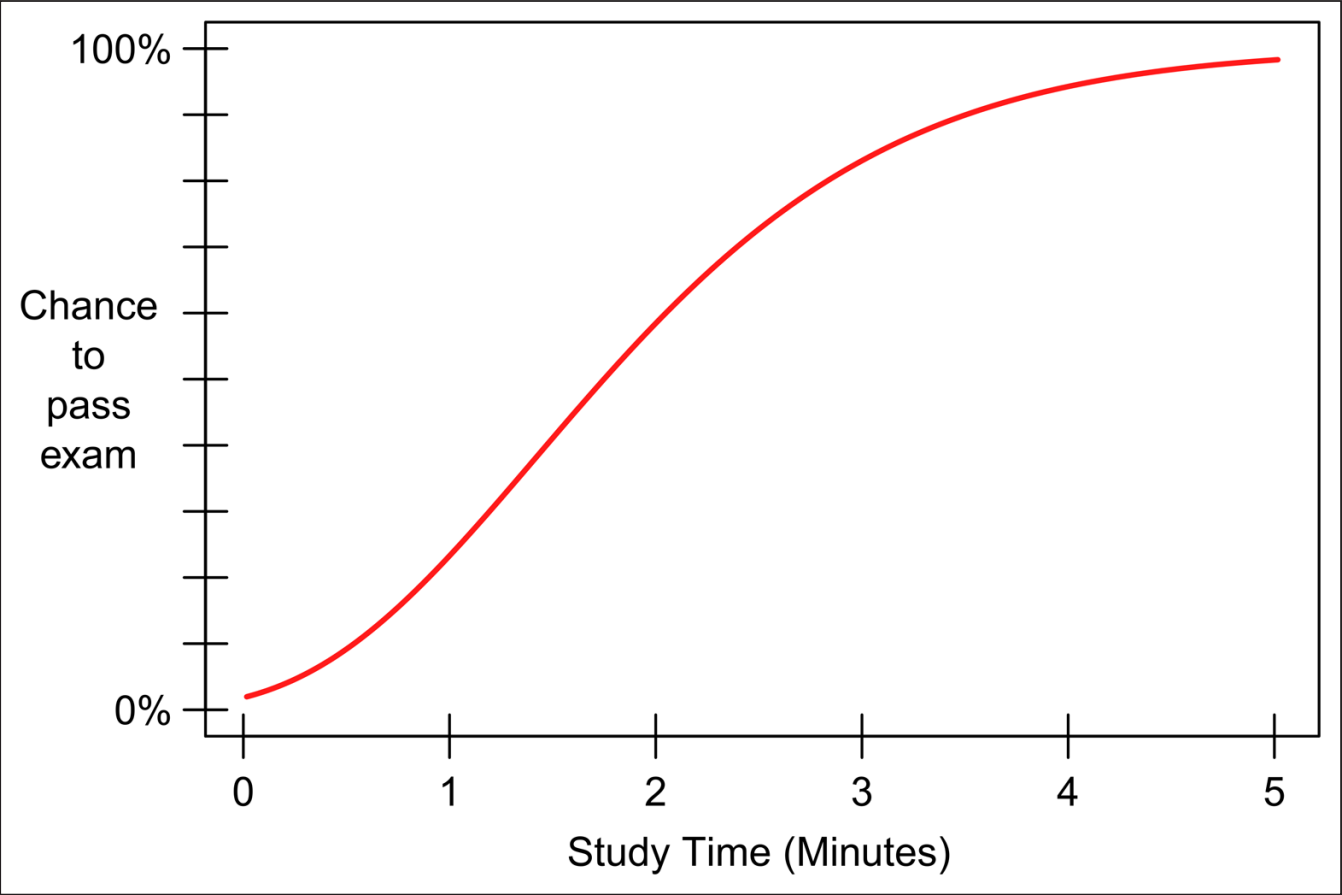
**Passing probability function.** The passing probability function used in Experiment 2.The function relates the investment of a maximum of 5 minutes of study time to the probability of passing an exam consisting of 10 3-alternative multiple-choice questions.

We adapted the PAL task used in Experiment 1 to include an indication of the probability that a participant would pass the test, given the amount of time they already spent studying at any point during the learning phase. Note that we could only provide a rough estimation of this probability, as it crucially depends on learning capability or learning speed, which is likely to show substantial variation both within and between individuals (e.g., Wang, 1983). To create this indication of passing probability, we used a computational model (Nijenkamp et al., 2016) to obtain a function (***Figure 2***) relating passing probability for an exam with ten 3-alternative multiple-choice questions and a passing grade of 6 out of 10 (no correction for guessing) to study-time investment, taking into account the maximum amount of time participants would be able to study the material in the actual experiment. The general form of the function, with relatively flat portions at very short and long study times, and an intermediate dynamic range of study times across which passing probability increases quite steeply from about zero to about one, is a characteristic of the highly nonlinear relationship between the amount of acquired knowledge and the passing probability for multiple-choice exams (for details, see Nijenkamp et al., 2016). This general form may therefore apply to all participants, and displaying a real-time indication of passing probability may provide them with useful information to help them estimate expected utility as well as motivate them to attempt to maximize it. The same hypotheses as in Experiment 1 were tested for Experiment 2.

### Method

#### Participants

The experiment was completed by 39 first-year students (18 female) from the Psychology Bachelor Program of the University of Groningen, who did not participate in Experiment 1. They participated in exchange for course credits. Their ages ranged from 18 to 27 (*M =* 20.5, *SD* = 2.1). The study was approved by the Ethical Committee Psychology (16177-S-NE), and participants gave their written informed consent prior to starting the experiment.

#### Materials and procedure

**Figure 3:**
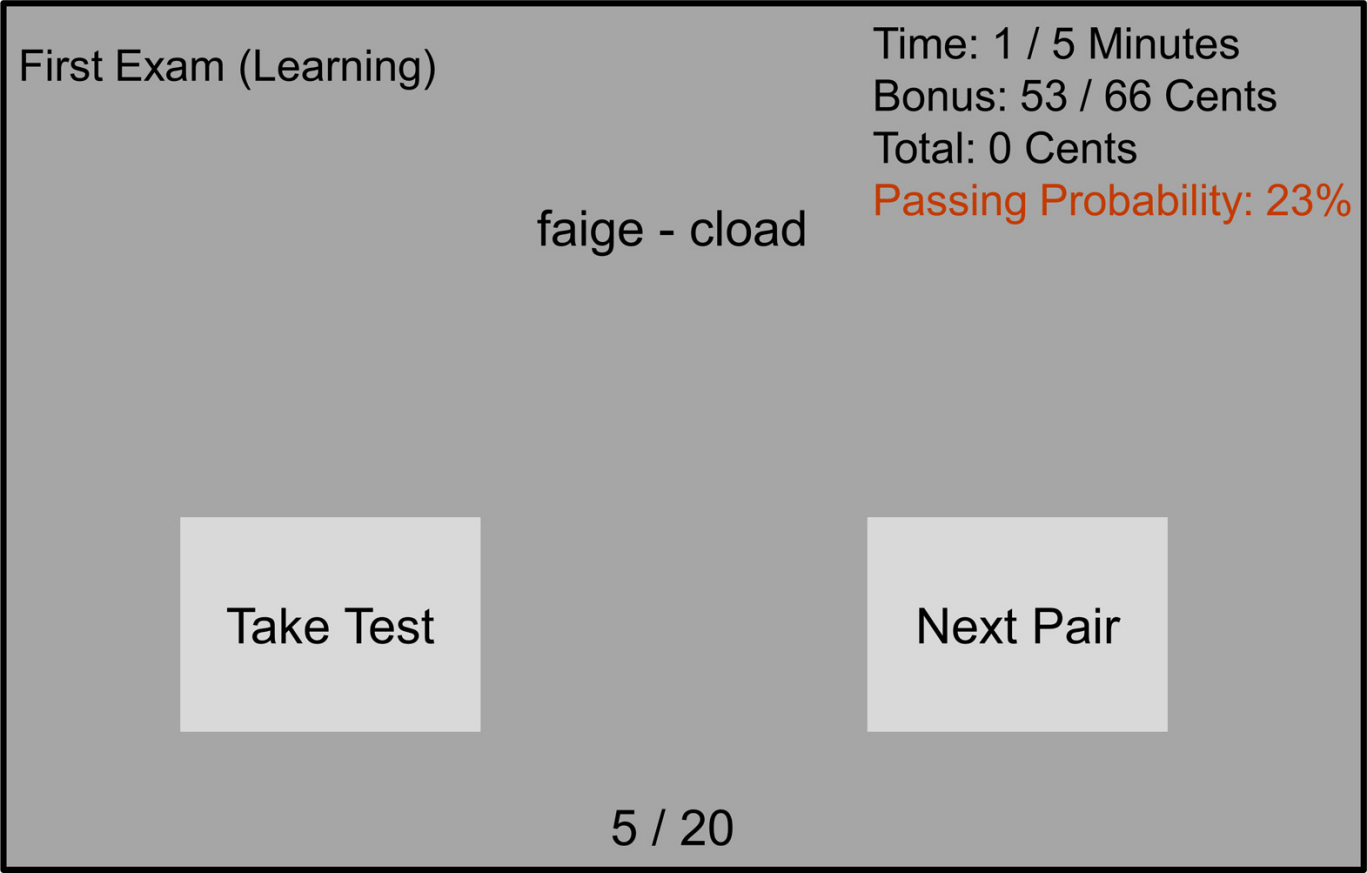
**Task display.** The task display as presented to participants during the study phase in Experiment 2. The display showed the pseudoword pair in the middle, an indication of the type of exam in the top left, an index indicating which word pair the participants was studying at the bottom, and the time, bonus, total earnings, and passing probability indication counters at the top right. Additionally, the display included two light-gray buttons on which the participants could click at any point to either continue to the next pair, or take the test.

The materials and procedure were the same as for Experiment 1, with the exception that in Experiment 2 an approximate passing probability (between 0% and 100%) as a function of invested study time was presented in the top-right of the screen, next to the invested time, the bonus counter, and the total bonus earned up until that point (***Figure 3***). The color of the passing probability approximation was initially red for all participants at the start of the learning phase, indicating a low passing probability, and became progressively greener as passing probability approached 100%. As explained above, the passing probability function was derived from a model of study-time investment on multiple-choice exams (Nijenkamp et al., 2016), assuming an exam consisting of ten 3-alternative questions, a passing grade of 6, and a maximum study time of five minutes (i.e., 300 seconds). The instructions that participants received were the same as in Experiment 1, with the addition of the following text that informed them about the function of the passing probability indication: “On the screen it will also state the passing probability for the exam when you are learning the word pairs. This passing probability is an estimate based on the amount of time you have spent during the learning phase, which you can use to make an informed decision about how much time you will spend on learning the word pairs”.

#### Data analysis

The data analysis procedure was the same as for Experiment 1.

### Results and discussion

To determine whether a resit effect occurred in Experiment 2, we compared the time participants spent studying the pseudoword pairs for R1 (*M* = 164.8, *SD* = 78.7) and NR (*M* = 168.3, *SD* = 72.7; ***Table 2***). A one-sided Bayesian paired samples t-test revealed there was substantial evidence (*BF*_10_ = 0.30) against the presence of a resit effect. A one-sided analysis examining the correlation between the resit effect (*M* = 3.5, *SD* = 34.5) and the CRT scores of participants showed substantial evidence (*r* = –.18, *BF*_10_ = 0.10) against our hypothesis of a positive correlation, indicating that the magnitude of the resit effect was not larger for more ‘rational’ participants.

**Table 2:** Descriptive Statistics Experiment 2.


TEST	MEAN OVERALL STUDY TIME IN SECONDS (*SD*)	MEAN STUDY TIME PER WORD PAIR IN SECONDS (*SD*)	MEAN NUMBER OF WORD PAIRS VIEWED (*SD*)	MEAN GRADE (*SD*)	MEAN PROPORTION OF PASSED TESTS (*SD*)

**NR**	168.3 (72.7)	5.9 (3.3)	31.0 (11.0)	7.1 (1.7)	0.74 (0.29)

**R1**	164.8 (78.7)	5.9 (3.1)	30.0 (10.3)	7.2 (1.8)	0.77 (0.30)

**R2**	101.2 (51.5)	4.3 (2.8)	25.3 (11.2)	6.9 (2.5)	0.81 (0.34)


*Note*: Mean values, averaged over both passed and failed tests, for overall study time in seconds, study time per word pair in seconds, number of word pairs viewed (values higher than the total amount of included word pairs per test reflect the repeated studying of certain pairs), grade, and percentage of passed tests for NR (single chance), R1 (first test opportunity), and R2 (resit opportunity) in Experiment 2. Standard deviations are presented in parentheses.

In accordance with the results of Experiment 1, a one-sided paired samples t-test revealed strong evidence (*BF*_10_ = 0.07) against our hypothesis that participants would spend less time per passed test in the resit condition (*M* = 192.2, *SD* = 75.8) than for the no-resit condition (*M* = 179.5, *SD* = 71.9).^4^ Another one-sided paired samples t-test revealed decisive evidence (*BF*_10_ = 24398) in favor of our hypothesis that the proportion of passed tests would be higher in the condition with resit opportunity, passed either on R1 or R2 (*M* = 0.94, *SD* = 0.19), than in the no-resit condition (*M* = 0.74, *SD* = 0.29).

As part of the exploratory analysis on the results of the surprise memory test, a two-sided Bayesian paired samples t-test revealed there was anecdotal evidence in favor of a null effect (*BF*_10_ = 0.43) indicating a failure to distinguish whether the proportion of correctly remembered word pairs did or did not differ between the no-resit (*M* = 0.6, *SD* = 0.2) and resit conditions (*M* = 0.7, *SD* = 0.2).

#### Meta-analysis of both experiments

Since the tasks used in Experiment 1 and 2 were nearly identical, with the exception of the added passing probability information for Experiment 2, we merged the data of both experiments for a combined analysis with greater statistical power. Using one-sided Bayesian paired samples t-tests to analyze the merged dataset, we found there was anecdotal evidence (*BF*_10_ = 0.51) against the hypothesis that time investments for R1 (*M* = 170.4, *SD* = 73.5) were lower than for NR (*M* = 175.3, *SD* = 70.4). We also found substantial evidence against the hypothesis of a positive correlation between the resit effect and the CRT (*r* = –.03, *BF*_10_ = 0.11). Additionally, we found strong evidence (*BF*_10_ = 0.05) against the hypothesis that less time would be invested per passed exam in the resit condition (*M* = 192.8, *SD* = 71.7) than in the no-resit condition (*M* = 170.4, *SD* = 73.5).^5^ Lastly, we found decisive evidence (*BF*_10_ = 61640) in favor of the hypothesis that the proportion of passed exams was higher in the resit condition (*M* = 0.94, *SD* = 0.17) than in the no-resit condition (*M* = 0.80, *SD* = 0.28).

## Discussion Experiments 1 and 2

In Experiments 1 and 2, we examined whether the prospect of a resit test would lead participants to reduce their actual study-time investment for a first test opportunity in a Paired-Associates Learning (PAL) task. The task required participants to spend time learning pseudoword pairs for a test under conditions with and without a resit opportunity. Despite our efforts to stay close to the design of the hypothetical study-time investment task that was previously found to produce robust and sizable resit effects (Nijenkamp et al., 2016, 2018), especially with the added passing probability indication in Experiment 2, the current study did not yield these effects in two experiments that involved the investment of actual time studying materials for a subsequent test. Furthermore, unlike the previous studies using the hypothetical study-time investment task, the current study did not yield support for a positive correlation of the resit effect with CRT scores and also did not replicate earlier findings suggesting that less study time may be invested per passed exam in a resit condition.

### Implications for the resit effect

Given the strikingly different behavioral effects of resit prospects in the hypothetical study-time investment task and the PAL task used in the above experiments, it is important to address what the absence of a resit effect with *actual* study-time investments might imply for the possible presence and underlying mechanisms of resit effects in learning scenarios requiring actual learning of materials. Initially, we thought of the need for actual time investments, as in the PAL task, as a better approximation of naturalistic learning scenarios than the rather abstract and artificial study-time investment task. Given this perspective, the absence of resit effects in the PAL task could therefore be taken to argue against the presence of resit effects in settings requiring actual learning. On the other hand, concerns about negative effects of a resit prospect in educational settings requiring actual learning have been expressed in the literature (Al-Bayatti & Jones, 2003; Burr et al., 2018; Centre for Education Research and Policy, 2012b, 2012a; Kickert et al., 2020; McManus, 1992; Non & Tempelaar, 2016; Pell et al., 2009; Ricketts, 2010; Scott, 2012; Yocarini et al., 2018), and have indeed been found in a field study by Grabe (1994; for similar findings, see Centre for Education Research and Policy, 2012b). Therefore, the notion of resit effects in settings requiring actual study time to be allocated to learning materials should perhaps not be dismissed just yet. Instead, we will argue that the PAL task could in fact have forced participants to approach their study-time investment in a different way than in the study-time investment task.

Two possible differences between the tasks could potentially underlie the discrepancy in the presence of a resit effect. Firstly, the PAL task did not ask for a priori study-time investment decisions. Instead, participants most likely made on-the-fly decisions regarding when to stop studying and take the test, based on their current judgment of learning (e.g., see Metcalfe & Kornell, 2005) and the information presented in the task. However, these on-the-fly decisions might be biased by illusions of competence that can occur when using paired-associates learning materials (Koriat & Bjork, 2005) and distorted timing judgments due to an increased cognitive load when studying the stimulus materials (e.g., see Block et al., 2010). Furthermore, the maximum monetary reward of €0.66 per test (maximum €4,- in total for all tests) might not have been sufficiently high to incentivize and motivate participants to perform well in the sense of optimizing the existing trade-off between the costs of investing study time by means of a lowered monetary reward and the benefits of passing the test by ultimately receiving the reward. This suggests that the nature of the study-time investment decision in the PAL task might not have enabled optimization behavior, and therefore did not produce a resit effect, despite all the necessary information being presented. In contrast, the study-time investment task only asked for an a priori decision with time for deliberation on the amount of hypothetical study time the participant would like to invest to have a certain probability of passing an exam, akin to making an advance study-time planning. Given the fact that the study-time investment task produced robust resit effects under differing conditions, the lack of a priori deliberation on the study-time investment decision in the PAL task could indeed be a factor that underlies the absence of a resit effect in Experiment 1 and especially Experiment 2.

The second difference between the tasks is that the study-time investment task asks for the investment of *hypothetical* study time to pass a simulated exam, whereas the PAL task required the investment of *actual* time to study materials for passing a short test. Studies have shown a decrease in risk taking when the consequences of a gamble are real as compared to hypothetical (e.g., Feather, 1959; Irwin et al., 1992; Lafferty & Higbee, 1974; Levin et al., 1988; Slovic, 1969; Xu et al., 2016). The fact that actual study-time investments were required in the PAL task could have led participants to not be willing to take the additional amount of risk on the first test opportunity (i.e. investing less time on studying) that the resit opportunity affords due to essentially being a ‘free do-over’, as re-sitting the test requires spending more time participating in the experiment. This suggests that perhaps the lack of a resit effect in the PAL task could be explained by the ‘real’, and thus likely more costly, consequences of study-time investment decisions.

## Experiment 3

To test the latter notion that perhaps it was the investment of *actual*, or real, time on studying materials that led to the absence of a resit effect in Experiments 1 and 2, we now turn to an adapted version of the original study-time investment task with which the resit effect was observed (Nijenkamp et al., 2016) and replicated (Nijenkamp et al., 2018). This adaptation introduced a condition requiring the investment of actual time, rather than solely hypothetical time. To associate the investment of hypothetical study time with the investment of a participant’s actual time, and thereby mimic a situation more similar to the investments made in the PAL task, we implemented a delayed feedback condition in the study-time investment task. Specifically, in Experiment 3 we compare the resit effect under two types of feedback: a condition with immediate feedback requiring only hypothetical time-investment decisions, as used in previous work, and a novel condition in which participants wait for feedback for an amount of time that depends on the amount of study time they invest. In essence, the current task should allow us to elucidate whether the resit effect is influenced by the nature of the time investment with which the investment decision is made. Furthermore, as previous work (Nijenkamp et al., 2016) has shown a positive correlation between the resit effect and an index of analytical/rational thinking (Cognitive Reflection Test, or CRT; see Frederick, 2005; also see Toplak et al., 2011), we included the CRT for this experiment as well (7-item version; Toplak et al., 2014).

We hypothesize that in the immediate-feedback condition we will observe the resit effect as previously found under identical conditions in the study-time investment task (Nijenkamp et al., 2016, 2018). With regards to the delayed-feedback condition, we hypothesize there will be no resit effect if the absence of the resit effect with the PAL task is related to the higher investment cost of ‘real’, rather than hypothetical, time investments.^6^ Alternatively, if the lack of a resit effect in the PAL task is related to the lack of an a priori, deliberated study-time investment decision we expect to observe a resit effect in the delayed-feedback condition. Furthermore, we expect any resit effects to be positively correlated with a participant’s CRT score (Toplak et al., 2014).

### Method

#### Participants

The a priori power analysis (Faul et al., 2007) for t-tests used for Experiments 1 and 2, assuming both a Type I and Type II error probability of .05 (i.e., power = 0.95) and based on the resit effect data from Experiment 1 by Nijenkamp and colleagues (2016; *M_NR_* = 6.22, *SD_NR_* = 0.53, *M_R1_* = 5.22, *SD_R1_* = 0.71, *r* = 0.58, *d_z_* = 1.69), revealed a necessary sample size of 6 participants to attain sufficient statistical power to observe a resit effect. Given this relatively low number of participants and the fact that Experiments 1 and 2 did not reveal a resit effect with real time investments, we decided to collect data from as many participants as possible within a single data collection period of two days to ensure sufficient statistical power for the delayed-feedback condition. Ultimately, the participant sample consisted of 65 first-year psychology students (54 female; *M_age_* = 19.7, *SD* = 2.1) from the University of Groningen, who participated for course credits. All participants gave their written informed consent prior to starting the experiment. Before data collection started, ethical approval was obtained from the Ethical Committee Psychology (17072-S-NE) of the University of Groningen.

#### Materials

**Figure 4:**
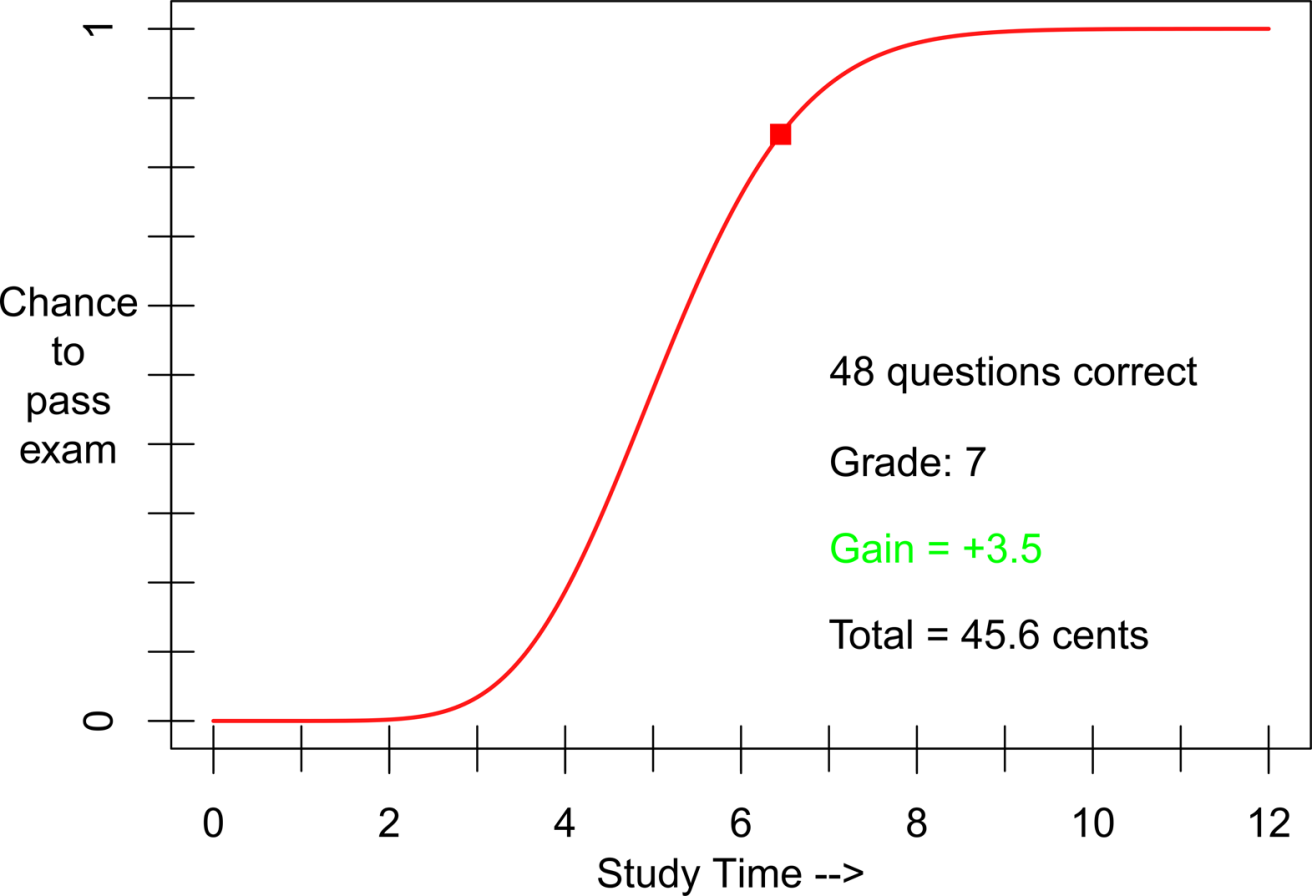
**Stimulus material.** The plot used as the stimulus material. Depicted is the relationship between hypothetical study time investment (x-axis) and the probability of passing a simulated exam (y-axis). Also depicted is the feedback, consisting of the outcome of the simulated exam, shown after participants invested their desired amount of hypothetical study time. Due to the arbitrary nature of the number of study-time units, the numbers on the x-axis were not presented to participants during the experiment, but are included in the figure for clarity.

The experiment was run in a lab consisting of 10 computer set-ups, enclosed by paperboard walls. We utilized the study-time investment task (Nijenkamp et al., 2016), programmed in MATLAB using the Psychophysics Toolbox extensions (Brainard, 1997; Kleiner et al., 2007). This task consisted of a graph with the probability of passing a simulated exam represented on the y-axis and study time, divided into 12 arbitrary units, represented on the x-axis (***Figure 4***). Participants could use the mouse to move a cursor along this function, and selected an amount of hypothetical study time by mouse click.

#### Design and procedure

Prior to starting the experimental task, participants completed the 7-item CRT (Toplak et al., 2014), found to be an index of rational-thinking tendencies (Toplak et al., 2011). Thereafter, participants received an instruction sheet detailing (1) the nature of the simulated exam, which was modeled after a multiple-choice exam consisting of 60 3-alternative questions and required a grade of 6 out of 10 to pass, (2) the probabilistic nature of the results of their study-time investment (i.e., investing the same amount of study time could lead to different exam outcomes), (3) the nature of the points system (i.e., each invested study-time unit cost one point, while passing the exam yielded 10 points), and (4) the general set-up of the task: one (no-resit condition) or two chances (resit condition) to pass the exam, with feedback received either immediately or after a delay that was proportional to the height of their investment. The delay in feedback was such that higher study-time investments equaled longer delays, with 800 milliseconds waiting time added per invested unit of study time. Practically, the average participant waited about 5 seconds per simulated exam.

In the No Resit (NR) condition participants had a single chance to pass the simulated exam, whereas in the resit condition participants were granted a resit opportunity (R2) to pass a failed first exam (R1). The feedback manipulation entailed that participants were given either immediate or delayed feedback. Due to the within-subjects nature of the study design, all participants completed all four possible conditions. The order in which these conditions were presented was counterbalanced across participants to account for potential order effects, with the restriction that the resit and no-resit conditions were always presented in alternation over the blocks. Half of the participants started with a resit opportunity, while the other half started with a single chance to pass the simulated exam. Participants always completed the single chance and resit blocks in succession within one feedback type before moving on to the other type of feedback. Half of the participants started the experiment with delayed feedback, while the other half started with immediate feedback. Taken together, each participant completed 4 blocks consisting of 30 trials (i.e., simulated exams) each. At the start of the first two blocks, participants completed 3 training trials to familiarize themselves with the task.

Each trial started with the presentation of the stimulus graph (***Figure 4***). Participants were instructed to move a cursor from the zero point along the presented function, and to click a mouse button to select the position on the function representing the amount of study time they wished to invest. After indicating their investment in the condition without delay, participants immediately received feedback about their exam score, the resulting grade, the number of lost or gained points, and their total points up to that moment in the task. In the delayed-feedback condition, feedback was presented after seeing the plotted function in the stimulus build up from the zero point to the point of the curve corresponding to their investment at a speed of 800 milliseconds per invested study-time unit – which made the waiting time, and therefore the ‘real’ consequences, dependent on their investment. The feedback remained on the screen for 2.5 seconds. In case of NR, participants progressed to the next trial/exam if they passed or failed. In the resit conditions, participants progressed to the next trial if they received a passing grade on R1. If they failed to receive a passing grade on R1 they progressed to the resit exam after the feedback for R1 was presented. Feedback for R1 also included whether or not a participant would have access to R2. R2 followed the same procedure as a NR trial.

#### Data analysis

The dependent variable used for data analysis consisted of the mean invested study-time units for both the NR and R1 exams in both the delayed- and immediate-feedback conditions. Data analysis was conducted using the JASP software package (Love et al., 2019). Bayes factors were used to assess the odds of our hypotheses being supported by the data (see Rouder et al., 2009). We used a Bayesian repeated-measures ANOVA (Rouder et al., 2012) and a Bayesian paired-samples t-test (Rouder et al., 2009) to assess whether the resit effect (i.e., the reduction in R1-time investments relative to NR investments) was present in both the immediate- and delayed-feedback conditions. Lastly, we used Bayesian correlation analyses to assess the degree to which the resit effect correlated to CRT scores.

Bayes factors were classified according to Wetzels and colleagues (2011), where Bayes factors (*BFs*) ≥ 3 and ≤ 10 or ≥ .1 and ≤ .33 are classified as ‘substantial’ evidence in favor of *H_1_* or *H_0_*, respectively, *BFs* between 10 and 30 or between .03 and .1 are classified as ‘strong’ evidence, *BFs* between 30 and 100 or between .01 and .03 are classified as ‘very strong’ evidence, and *BFs* > 100 or < .01 are classified as ‘decisive’ evidence. Additionally, *BFs* between 1 and 3 or between 0.33 and 1 are classified as ‘anecdotal’ evidence in favor of *H_1_* or *H_0_*, respectively.

##### Outlier exclusion

Investments of less than 2 or more than 10 study-time units were considered outliers and these trials were excluded from our analyses. This entailed a loss of 1.1% of all recorded trials. A comparison of the results with and without these outliers showed that their exclusion did not lead to any noteworthy differences in results.

### Results

**Figure 5:**
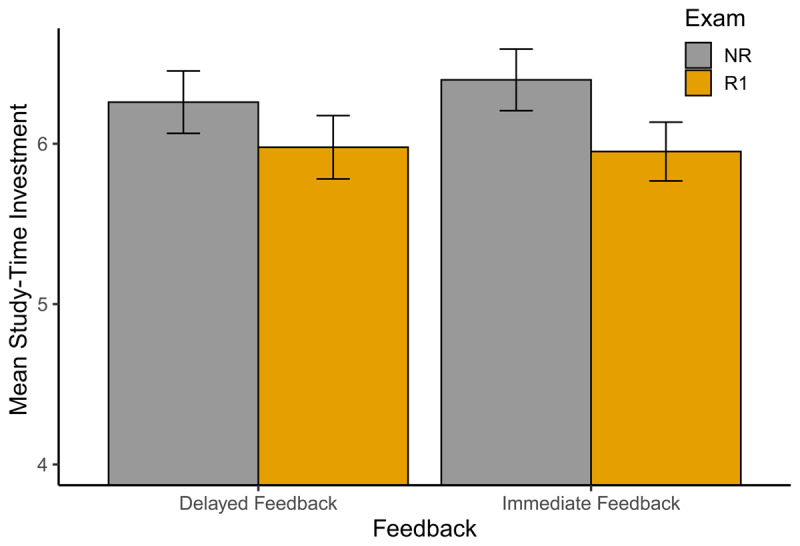
**Mean study-time investment.** Mean study-time investments for NR (single exam) and R1 (first exam with resit opportunity), per feedback condition (immediate vs. delayed). Error bars represent the 95% confidence intervals for the means.

A Bayesian repeated measures ANOVA revealed that the study-time investment data were best described by a model including just the main effect of exam type (i.e. NR or R1; see also ***Figure 5***). An analysis of effects using the inclusion Bayes Factor across matched models (Mathôt, 2017) revealed there was decisive evidence in favor of the inclusion of the main effect of exam type (*BF_incl_* = 6.35 × 10^8^), whereas there was anecdotal evidence against the inclusion of the main effect of feedback type (*BF_incl_* = 0.39) and the interaction between both main effects (*BF_incl_* = 0.39). As the ANOVA results did not show support in favor of the main effect for feedback type nor the interaction effect, the data for both the NR and R1 exam-types were collapsed over the feedback conditions for further analyses. A Bayesian paired samples t-test revealed decisive evidence (*BF_10_* = 4.14e^5^) in favor of R1-investments (*M* = 5.97, *SD* = 0.74) being lower than NR-investments (*M* = 6.33, *SD* = 0.75), showing that participants invested less time when a resit option was available (i.e., the resit effect).

To test whether the resit effect (NR investments minus R1 investments) based on these collapsed NR and R1 variables was positively correlated with a participant’s CRT score, we conducted a Bayesian correlation test. The test revealed substantial evidence (*r* = .31, *BF_10_* = 6.82) in favor of a positive correlation between the resit effect (*M* = 0.36, *SD* = 0.48) and CRT scores (*M* = 2.60, *SD* = 1.94).

## Discussion Experiment 3

With Experiment 3 we aimed to assess whether the investment of actual time, rather than merely hypothetical time, would reduce the magnitude of the resit effect (see Nijenkamp et al., 2016) to such a degree that it would be absent, or at the very least reduced in magnitude. Using Bayesian analyses, we replicated the resit effect reported in previous studies using the study-time investment paradigm with hypothetical time investments (i.e., immediate feedback; Nijenkamp et al., 2016, 2018). Furthermore, we also replicated the finding that the magnitude of the resit effect is correlated positively with a participant’s CRT score, a measure of analytical/rational thinking (Nijenkamp et al., 2016; see also Toplak et al., 2014). These findings once again reinforce the idea that participants in the study-time investment task are able to optimize their investments, possibly by approximating the shifting maximum expected utility associated with the trade-off between the costs of investing hypothetical time and the benefits of passing the simulated exam under differing resit conditions.

In Experiment 3 we also introduced a novel condition in which participants invested actual time, by delaying the feedback they received with an amount of time that was proportional to their study-time investments. As no resit effect was observed in Experiments 1 and 2 where participants solely invested actual time on studying for and making a test, the aim of this manipulation was to assess whether the magnitude of this effect would be affected by the inclusion of actual time investments in the study-time investment task. The data did not support this hypothesis, however, suggesting that the lack of a resit effect when using the Paired Associates Learning (PAL) task paradigm might not have been due to participants having to invest real time.

### Implications for the resit effect

The finding that feedback type did not affect the resit effect suggests that the nature of the time investment (i.e., actual or hypothetical) might not influence the effect. On the other hand, the results of Experiment 3 (***Figure 5***) do provide an indication, though admittedly rather weak, for a reduced resit effect with delayed feedback, and thus with the investment of actual time. Such a reduction would follow the predictions of study-time investment models (e.g., see Nijenkamp et al., 2016) indicating the resit effect would decrease in magnitude as the investment cost per unit of study time increases.^7^ If investing actual time was indeed experienced as more costly than investing hypothetical time, it stands to reason that the delayed-feedback condition did show a resit effect because the investment possibly was not sufficiently costly to approach the costs induced with the investment of actual time in learning the stimuli of the PAL task. Additionally, having to wait in the delayed-feedback condition might not have been perceived as costly, or ‘real’, enough to reduce the risk-taking tendencies that a resit opportunity seems to promote. This implies that the results of the current experiment could have been somewhat different if the waiting time per invested study-time unit would have been long enough to induce a sufficiently high experienced investment cost.

A different implication of the current results is that they support the view that the PAL task produced no resit effect due to the fact that study-time investment decisions were made on-the-fly, and therefore were not based on planned a priori deliberation. To illustrate, even if we assume that participants in the PAL task were in fact willing, motivated, and capable to take more risk in a resit scenario by studying less for a first-exam chance (as suggested by previous studies using the study-time investment task; Nijenkamp et al., 2016, 2018), ultimately they had to simultaneously invest actual time on studying to pass a test and optimize those investments according to utility maximization logic at the same time. As a result, participants might have opted instead to simply complete the apparent main task of studying items for a test and did not complete the seemingly secondary goal of optimizing their time investments. Since the study-time investment task used in Experiment 3 did not include the requirement of studying materials for a subsequent test, but simply asked for the investment of hypothetical or actual time to pass a simulated exam, participants still retained the opportunity to make a deliberated a priori decision to maximize utility, given the costs of investing study time and the gains associated with passing. As a result, participants produced a resit effect in the delayed-feedback condition as well.

The occurrence of the resit effect only when there is room for a priori deliberation implies that such an effect might occur in students’ actual study-time planning as well. Study-time allocation by means of advance strategic planning of multiple competing and time-consuming activities might form a plausible mechanism underlying study-time allocation strategies (e.g., see Ariel et al., 2009; Puustinen & Pulkkinen, 2001; Zimmerman, 2002). Especially in the context of higher education, where there is a marked emphasis on self-regulated learning, such strategic planning strategies might indeed be utilized. Study-time allocation strategies are found to be influenced by a number of factors, such as judgments of item difficulty, judgments of one’s own learning abilities, and learning goals (Cull & Zechmeister, 1994; Dunlosky & Hertzog, 1998; Metcalfe, 2009; Metcalfe & Finn, 2008; Metcalfe & Kornell, 2005; Son & Metcalfe, 2000; Thiede & Dunlosky, 1999). We suggest therefore that study-time allocation strategies are influenced by the prospect of a resit exam as well. If so, the resit effect might be related to students’ changing decision rules when making a study-time planning, given either one or two chances to pass an exam; a situation that should afford the time and mental space to apply utility maximization principles to optimize their learning (see Son & Sethi, 2006). Assuming that, at least part of, an advance study-time planning translates to actual study-time investment behavior as part of a self-regulated learning approach (Van Den Hurk, 2006; see also Puustinen & Pulkkinen, 2001; Zimmerman, 2002), we propose that the reported negative effects of a resit prospect on first-exam performance in naturalistic study environments (Centre for Education Research and Policy, 2012b; Grabe, 1994) reflect changes in students’ study-time allocation strategies when making such a study planning.

## Questionnaire Study

To support the notion that a resit opportunity might indeed affect advance study-time allocation strategies, we additionally discuss the results of a questionnaire study in which students were asked about their study behavior. More specifically, two questions asked whether students would study more, less, or the same for two previously completed exams if they had known in advance that the resit exam was not an option for them. We expect that students would indeed indicate that they would spend more time preparing for their exam if the option to resit would not have been available. For this hypothesis we follow the results from the studies using the study-time investment task (Nijenkamp et al., 2016, 2018), as the study time involved in this questionnaire is hypothetical in nature rather than real in the sense of the PAL task. To further contextualize the results for these main questions, we also report the results of additional questions assessing the amount of time participants actually spent in preparation for the exams and whether they ultimately passed the corresponding courses.

### Method

#### Participants

In total, 92 participants completed the questionnaire in return for course credits. Of these participants, five were excluded due to having indicated on the last question of the survey that they did not provide truthful answers. Ten participants were excluded from the dataset as they did not complete the entire survey. Finally, five participants were excluded for answering the questionnaire in less than five minutes (300 seconds), while several test runs had shown it takes at least five minutes to finish the entire survey, Therefore the data from these participants was regarded as potentially dubious. Of the remaining 72 participants (44 female), 48 indicated their age was between 18 and 20, and the remaining 24 participants indicated their age was above 20. Before data collection, the study was approved by the Ethical Committee Psychology (PSY-1819-S-0058) of the University of Groningen. Additionally, participants gave their written informed consent prior to filling out the questionnaire, and were debriefed about the purpose of the study after completion.

#### Materials and procedure

The questionnaire consisted of questions taken from scales assessing, the academic delay of gratification (Bembenutty & Karabenick, 1998), personality traits (Soto & John, 2017), academic self-efficacy (Davidson et al., 2009), and time management behavior (Macan et al., 1990). Furthermore, the questionnaire contained questions regarding students’ study habits for specific courses of their first-year psychology curriculum. The main questions of relevance for this study asked participants to indicate whether they would ‘*study more*’, ‘*study the same*’, or ‘*study less*’ in response to the following scenario and question about two courses for which they completed the exam previously: ‘*Imagine you have planned your vacation and you are very excited to go. At the beginning of the block you check the exam schedule and you find out that the resit of Developmental/Social Psychology got rescheduled. The resit is now planned during your vacation. Unfortunately, you cannot change your vacation plans. If this would have actually happened to you, what would you do*?’ Another question assessed how much time participants had actually spent in preparation for the completed exams of these two courses (*‘How much time did you put into the preparation of the Developmental/Social Psychology exam?’*), on which they could answer either ‘*Very little, so I did not expect to pass the exam*’, ‘*Just enough to hopefully pass the exam*’, ‘*A good amount that would ensure me of passing the exam*’, or ‘*A lot, so that I could obtain a high grade*’. A final question, to assess whether participants ultimately passed the respective exams, asked ‘*Did you pass the Developmental/Social Psychology exam?*’ on which they could answer either ‘*Yes*’ or ‘*No*’.

##### Educational context

The questionnaire was completed by first-year students at the University of Groningen enrolled in the English and Dutch Bachelor programs of Psychology. The first year of the program consists of 11 courses (worth 60 ECTS) of seven weeks, and is divided into four blocks of approximately two months each. Exams take place at the end of each block, and in the year of data collection the resit exams were scheduled after the exam period of the next block. The questionnaire asked students about their study habits for two courses (i.e., Developmental- and Social Psychology) that were assessed through a multiple-choice exam on which students were graded on a 10-point scale (1 = poor, to 10 = perfect). The minimum grade required to pass the exam was 5.5. If students did not obtain a passing grade, they had the option to resit their failed exam. There were no restrictions on the grade for the resit exam, except for the fact that if a student resits a failed exam their final grade will be the last grade they obtained.

### Results and discussion

The JASP software package (Love et al., 2019) was used to compute Bayesian multinomial tests to analyze the questions regarding whether students would invest more, less, or the same amount of time on studying for a first exam opportunity if they knew they could not attend the resit opportunity for each exam. For the Social Psychology exam, decisive evidence (see Wetzels et al., 2011) was found for the observed pattern of answers to be different than a null model using a uniform answer distribution (*BF_10_ =* 6.97 × 10^20^) and a model in which the majority of participants (*n =* 70) would have answered they would study the same when there was no option to resit (*BF_10_ =* 1.65 × 10^106^). For the question regarding Developmental Psychology, decisive evidence was found as well for the observed pattern of participants’ answers to be different than a uniform answer distribution (*BF_10_ =* 6.15 × 10^14^) and a model where the majority of answers (*n* = 70) were they would have studied the same (*BF_10_ =* 6.27 × 10^82^). Taken together with the frequency distribution data (***Table 3***), the above analyses seem to conclude that, if the students knew in advance there was no resit exam, most participants would have studied more than if the option to resit was available. This conclusion seems to imply that students take the availability to resit a failed exam into account when deciding on how much time to allocate to studying for an exam.

**Table 3:** Frequency Distribution Table.


COURSE	DISTRIBUTION OF ANSWERS (%)		

	‘STUDY LESS’	‘STUDY THE SAME’	‘STUDY MORE’

**Social Psychology**	0 (0%)	8 (11.1%)	64 (88.9%)

**Developmental Psychology**	0 (0%)	17 (23.6%)	55 (76.4%)


*Note*: Frequencies, with proportions in parentheses, of the number of participants that answered ‘study less’, ‘study the same’, or ‘study more’ to questions about two courses (Social Psychology and Developmental Psychology) for a questionnaire study that asked about how participants’/students’ study habit for an exam would have changed if they knew in advance they would have no option to resit in case of failure.

Using JASP, we analyzed two other questions of interest using Bayesian contingency tables, with the ‘sample’ parameter set to a joint multinomial due to the sampling method used to collect the data (see Jamil et al., 2017). The Bayesian contingency table produces a Bayes factor that indicates the evidence in favor of the alternative hypothesis of dependence between rows and columns (or against *H_0_*, independence between rows and columns). In the context of the current analysis, the rows signify the amount of exam preparation a student indicated on the questionnaire, and the columns represent whether a student indicated they passed the exam (***Tables 4*** & ***5***). The analyses revealed decisive (*BF_10_* = 317) and very strong (*BF_10_* = 58) evidence in favor of the alternative hypothesis of dependence between rows and columns, for both the Developmental- and Social Psychology exams, respectively. Turning to ***Tables 4*** and ***5***, this suggests that those who indicated they studied more indeed were more likely to obtain a passing grade as well. This might seem unsurprising; however, it suggests that the students’ metacognitive judgments regarding their study-time investment do indeed seem accurate enough to provide some insight into their actual study behavior.

**Table 4:** Contingency Table Developmental Psychology Exam.


*‘HOW MUCH TIME DID YOU PUT INTO THE PREPARATION OF THE EXAM?’*	*‘DID YOU PASS THE EXAM?’*		

	NO	YES	TOTAL

**‘A lot, so that I could obtain a high grade’**	0	12	12

**‘A good amount that would ensure me of passing the exam’**	6	26	32

**‘Just enough to hopefully pass the exam’**	7	16	23

**‘Very little, so I did not expect to pass the exam’**	5	0	5

**Total**	18	54	72


**Table 5:** Contingency Table Social Psychology Exam.


*‘HOW MUCH TIME DID YOU PUT INTO THE PREPARATION OF THE EXAM?’*	*‘DID YOU PASS THE EXAM?’*		

	NO	YES	TOTAL

**‘A lot, so that I could obtain a high grade’**	0	8	8

**‘A good amount that would ensure me of passing the exam’**	8	15	23

**‘Just enough to hopefully pass the exam’**	12	14	26

**‘Very little, so I did not expect to pass the exam’**	11	4	15

**Total**	31	41	72


Finally, the question regarding whether the students would have changed their study-time investment if they knew the resit exam would not be available for them, and the question about how much they had actually studied were investigated using Bayesian contingency tables as well (***Tables 6*** & ***7***). The analyses revealed only anecdotal evidence against the alternative hypothesis for the Developmental- (*BF_10_* = 0.41) and Social Psychology (*BF_10_* = 0.30) exams, indicating evidence in favor of independence between the two questions. Upon inspection of ***Tables 6*** and ***7***, however, one can see this seems to be the case because most students indicated they would study more for their first exam chance if they knew the resit would not be available, regardless of how much study time they indicated they had already invested. This shows that even those who indicated they already studied a lot would aim to study even more if the resit would not have been available.^8^ Ultimately, this suggests that the availability of a resit exam does indeed influence the amount of time that is planned to be spent studying for an exam.

**Table 6:** Contingency Tables Developmental Psychology Exam.


*‘HOW MUCH TIME DID YOU PUT INTO THE PREPARATION OF THE EXAM?’*	*‘IMAGINE YOU HAVE PLANNED YOUR VACATION…’*		

	‘STUDY MORE’	‘STUDY THE SAME’	TOTAL

‘A lot, so that I could obtain a high grade’	8	4	12

‘A good amount that would ensure me of passing the exam’	22	10	32

‘Just enough to hopefully pass the exam’	20	3	23

‘Very little, so I did not expect to pass the exam’	5	0	5

**Total**	55	17	72


**Table 7:** Contingency Tables Social Psychology Exam.


*‘HOW MUCH TIME DID YOU PUT INTO THE PREPARATION OF THE EXAM?’*	*‘IMAGINE YOU HAVE PLANNED YOUR VACATION…’*		

	‘STUDY MORE’	‘STUDY THE SAME’	TOTAL

‘A lot, so that I could obtain a high grade’	5	3	8

‘A good amount that would ensure me of passing the exam’	21	2	23

‘Just enough to hopefully pass the exam’	24	2	26

‘Very little, so I did not expect to pass the exam’	14	1	15

**Total**	64	8	72


## General Discussion

While the lack of resit effects with the Paired-Associates Learning (PAL) task used in Experiment 1 and the adapted version including an indication of passing probability in Experiment 2 shows there are boundaries to the conditions under which such effects occur, it does not necessarily imply there are no resit effects possible in learning scenarios requiring the investment of actual time on studying materials for a subsequent test. In fact, together with the presence of the effect in Experiment 3 under conditions requiring some investment of actual time and the fact that the questionnaire study showed that university students indicated they would have indeed studied more for an actual exam if the option to resit that exam would not have been available, the overall results suggest that a resit prospect may indeed primarily affect advance study-time allocation strategies (i.e., making a study planning). If this is the case, then the normative allocation of hypothetical study time in the investment task and accompanying model (Nijenkamp et al., 2016) can be taken as a proxy of advance or planned allocation of study time. The similarity between the mechanisms of the investment task and advance study-time allocation strategies is further emphasized by the fact that the relevant tradeoffs between costly study-time investments and probabilistic benefits of successfully passing exams in the investment task seem similar to those guiding the advance allocation of limited, hence costly, study time across multiple exams, or multiple learning goals, in real-life academic settings. In both cases the goal is to identify optimal study-time investments or allocation policies that maximize expected utility. Also common to both cases is the rather abstract and hypothetical nature of the time investments and consequences under consideration, which, as discussed previously, has been shown to enhance people’s willingness to take risks such as those involved in the resit effect.

A potentially significant difference between the study-time investment task, that has produced resit effects, and real-life study scenarios, however, is that participants in the investment task were provided with explicit and precise graphical information about passing probability as a function of invested study time. In real life, on the other hand, students undoubtedly must deal with less precise information about this relationship (Bjork et al., 2013). However, the resit effect might not critically depend on such precise and detailed information; it only requires students to appreciate the rather obvious fact that passing probability smoothly increases as a function of study time, and that the prospect of a resit offers them an almost risk-less opportunity to attempt to pass the first exam with considerably less invested study time than they would have invested without a resit opportunity. This latter appreciation is clearly indicated, especially for more rational students, by the robust resit effects that have previously been obtained with the study-time investment task, and in part by the results of the current questionnaire study that seem to indicate students would indeed study more when the resit exam would not have been available.

All in all, from these considerations the presence of resit effects in students’ advance study-time allocation and planning decisions would seem possible, and indeed plausible. Note that, assuming students stick to these advance study-time allocation plans (Van Den Hurk, 2006; see also Puustinen & Pulkkinen, 2001; Zimmerman, 2002), this would virtually guarantee a corresponding resit effect in the actual study time invested in preparing for an exam with a resit prospect. To illustrate, imagine a student planning their finals week and allocating five days for final preparations preceding an exam with no resit opportunity, but only three days in case of an exam with a resit opportunity to accommodate this timing conflict between academic pressures (see Grabe, 1994). This resit effect in the advance planning would almost surely translate to a similar effect in actual study-time allocation. Even in the case that the student would not strictly adhere to their preconceived study planning, one could imagine, based on the results of the questionnaire study, they would still appreciate the fact that having a resit option for the one exam allows them to prioritize their investment of limited study time for the exam without the resit opportunity, rather than studying equally for both. Essentially, the option to resit an exam might signal a lower-cost opportunity to optimize the trade-off between the benefits of passing the exam and the costs of investing limited time on studying, thereby allowing for more ‘risky’ study-time allocation strategies that yield a lower chance of passing but require less study-time investment.

## Data Accessibility Statement

Data can be accessed at *https://doi.org/10.17605/OSF.IO/XV4S7*.

## Footnotes

As a case in point, across different bachelor and masters programs in Universities in the Netherlands, different resit policies are implemented, with some not offering any resits, highlighting the lack of knowledge and consensus about the effects of resit exams.

In a pilot study, the use of English words in the PAL task proved to be too easy for participants. This caused test performance to be close to ceiling across all conditions with little study-time investment, and left participants with little room to possibly reduce their study-time investments as part of the experimental manipulation.

A passed exam in the resit condition is an exam that is passed through either R1 or R2. As the particular analyses in this paragraph only include data from passed tests, the descriptive statistics for the resit and no-resit conditions reported here deviate from those reported in ***Table 1***. Additionally, two participants, who did not pass any NR tests, were excluded from this analysis. Excluding these participants from the main analyses yielded no noteworthy differences in results.

As the particular analyses in this paragraph only include data from passed tests, the descriptive statistics for the resit and no-resit conditions reported here deviate from those reported in ***Table 2***. Additionally, two participants, who did not pass any NR tests, were excluded from this analysis. Excluding these participants from the main analyses yielded no noteworthy differences in results.

Two participants, who did not pass any NR tests, were excluded from this analysis. Excluding these participants from the main analyses yielded no noteworthy differences in results.

More recent simulations of our model of study-time investment (see Nijenkamp et al., 2016) indeed predict that the resit effect should become smaller when the costs associated with investing a unit of study time are higher.

Interestingly, with higher investments costs the model predicts a lower optimal point for NR study-time investments than for R1 investments. In Figure 5 it can be visually assessed that participants indeed show an indication of this, as they invested less on NR in the delayed-feedback condition (*M* = 6.26, *SD* = 0.80) than in the immediate-feedback condition (*M* = 6.40, *SD* = 0.79; *BF_10_* = 2.55), and that this reduction was not as large for R1 investments with delayed (M = 5.98, SD = 0.81) versus immediate feedback (*M* = 5.95, *SD* = 0.75; *BF_10_* = 0.10). Since these differences between NR and R1 investments over feedback conditions were not fully statistically supported, we can only say that the data followed the model predictions in a qualitative manner. This points to the fact that this model might overestimate the magnitude of the predicted effects (see also Nijenkamp et al., 2016, 2018).

Using the model of study-time investment by Nijenkamp and colleagues (2016), the resit effect (i.e., a reduced study-time investment due to the prospect of a resit exam) is indeed predicted to also be present, albeit with reduced magnitude, for those students that aim to achieve high grades rather than merely passing with a minimal grade.

## Author Information

Rob Nijenkamp is now at Educational Support and Innovation –Team Educational Innovation & Evaluation, Center for Information Technology, University of Groningen.
